# Ethyl Acetate Fraction from a *Catalpa ovata G*. Don Extract Inhibits ɑ-MSH-Induced Melanogenesis through the cAMP/CREB Pathway

**DOI:** 10.3390/ijms25010151

**Published:** 2023-12-21

**Authors:** Yon-Suk Kim, Eun-Bin Lee, Ye-Ji Yu, Ga-Won Kim, Woo-Jung Kim, Dong-Kug Choi

**Affiliations:** 1 Department of Biotechnology, Research Institute of Inflammatory Disease (RID), College of Biomedical and Health Science, Konkuk University, Chungju 27478, Republic of Korea; 2 Department of Applied Life Sciences, Research Institute (RIBHS), College of Biomedical & Health Science, Graduate School, Konkuk University, Chungju 27478, Republic of Korea; 3 Biocenter, Gyeonggido Business and Science Accelerator, Gwanggyo-ro 147, Yeongtong-gu, Suwon 16229, Republic of Korea

**Keywords:** *Catalpa ovata*, tyrosinase, MAPK, melanogenesis, cAMP/CREB

## Abstract

The whitening effect of reducing skin pigmentation is one of the most important goals of cosmetics. The purpose of this study was to determine whether *Catalpa ovata* extract and its fractions have potential as natural skin-lightening agents. Initially, we screened various fractions of *Catalpa ovata* extract using an in vitro antioxidant assay. Then, the inhibitory effects of *C. ovata* extract and its fraction on melanogenesis and the related mechanisms were investigated in B16F1 melanoma cells. The results showed that the ethyl acetate fraction (EF) from *C. ovata* extract markedly inhibited melanin synthesis in a dose-dependent manner at non-toxic concentrations. Furthermore, EF downregulated both the protein and mRNA levels of tyrosinase, which is a specific enzyme that catalyzes the conversion of tyrosine into melanin. We also found that EF decreased the microphthalmia-associated transcription factor (MITF) at the protein and mRNA levels. EF increased the phosphorylation of ERK and suppressed the phosphorylation of JNK and p38 in ɑ-MSH-induced B16F1 cells. These results indicate that EF can regulate the MAPK pathway. In addition, EF has an anti-melanogenic effect via the downregulation of intracellular cyclic-AMP (cAMP). Nineteen major compounds of EF were identified using LC-MS/MS. Taken together, these results suggest that EF may be a potential anti-melanogenic agent for use in skin-whitening cosmetics and in topical treatments for hyperpigmentation disorders.

## 1. Introduction

Melanin is the major pigment synthesized by the epidermal melanocytes of the skin [1]. At the cellular level, melanin synthesis occurs in highly specialized organelles and is regulated by precise mechanisms that include organelle formation, the synthesis and transfer of enzymes, structure and regulation proteins and cofactors, substrates, and copper, and the dynamic transformation and speed of the final activation and processes [2]. One of the most important functions of melanin is the physiological defense that protects the skin from ultraviolet (UV) radiation [3]. However, repeated exposure to UV radiation can cause problems such as melanin accumulation, skin hyperpigmentation, wrinkles, and skin cancer [4]. Therefore, the inhibition of melanogenesis is one way to solve skin problems, so the study of the mechanism of inhibition of melanogenesis is of major interest in the pharmaceutical and cosmetic fields. 

cAMP synergists, such as α-melanocyte-stimulating hormone (α-MSH), isobutyl-methylxanthine, forskolin, and adrenocorticotropic hormone, are known to induce melanogenesis [5]. A-MSH is widely used as a substance that induces melanin production in in vitro models [6]. The binding of α-MSH to its receptor, melanocortin-1 receptor (MC1R), induces the expression of microphthalmia-associated transcription factor (MITF) and the cAMP response element binding protein (CREB), leading to the increased expression of tyrosinase and other melanogenesis-related enzymes, such as tyrosinase-related protein 1 (TRP-1) and tyrosinase-related protein 2 (TPR-2) [7]. Therefore, many studies on the regulation of melanogenesis have used α-MSH as a positive inducer [8]. Tyrosinase is a copper-containing glycoprotein that is important in melanin synthesis [9]. In melanin biosynthesis, tyrosinase is a bifunctional enzyme that modulates melanin production—first by catalyzing the hydroxylation of L-tyrosine to L-3,4-dihydroxyphenylalanine (L-DOPA) and, secondly, by catalyzing the oxidation of L-DOPA to DOPA quinone [10]. TRP-2, which acts as a dopachrome tautomerase, changes dopachrome into 5,6-dihydroxyindole-2-carboxylic acid (DHICA) [11,12]. TRP-1 oxidizes DHICA into a carboxylated indole-quinone, which eventually facilitates the generation of melanin [13].

MITF is a master transcription factor that can transcriptionally regulate melanogenic processes. MITF is regulated by various signaling pathways, one of which is known to be regulated by mitogen-activated protein kinase (MAPK) signaling. MAPKs are a type of protein kinase specific for serine and threonine and are known to play an important role in regulating melanogenesis and regulating cellular functions such as cell differentiation, proliferation, mitosis, apoptosis, and cell survival [14]. One member of the MAPK family, ERK, has been reported to phosphorylate MITF at its 73rd serine residue upon activation of ERK through c-Kit stimulation. In addition, the phosphorylation of MITF at the 73rd serine is followed by MITF ubiquitination and degradation [15]. The degradation of MITF decreases the expression of tyrosinase and other melanogenic enzymes, which, in turn, leads to the inhibition of melanin synthesis. Moreover, JNK, the p38 signaling pathway activator, could also increase melanogenesis by inhibiting tyrosinase activity [16].

Several melanogenesis inhibitors have been reported, including arbutin, kojic acid, and linoleic acid. Kojic acid and arbutin are widely used as cosmetic ingredients because of their anti-tyrosinase activity [17]. Kojic acids have some benefits and risks. The advantages of kojic acid as a lightening agent are the lightening effect on visible sun damage and age spots, anti-aging outcomes, and anti-acne properties [18,19]. The disadvantages of kojic acid are contact dermatitis (especially for sensitive skin), as well as skin irritation, itching, rashes, and pain. Kojic acid could also result in skin cancer on damaged skin [20]. So, we set out to find potential new compounds from natural sources with few side effects. Therefore, the current study aims to investigate compounds that are inhibitory of melanogenesis and that are from natural sources.

*Catalpa ovata G*. Don (*C. ovata*) is widely distributed in Korea and China and has been used in folk remedies for a long time, and the fruits of *C. ovata* have been used as a diuretic in the treatment of chronic nephritis and edema [21]. In experimental studies, the anti-microbial, antitumoral, and anti-inflammatory effects of the stem bark of *C. ovata* have been reported [22,23,24]. However, there have not been any further investigations of the inhibitory potential of *C. ovata* for melanogenesis.

The purpose of this study was to investigate the possibility of the use of *C. ovata* extract as a whitening agent. To the best of our knowledge, this study is the first to reveal the beneficial anti-melanogenic effects of *C. ovata* and EF against α-MSH-stimulated melanogenesis responses in B16F1 cells. The *C. ovata* water extract was divided into five fractions, and the superior fraction was selected by measuring their effects on the inhibition of melanin production. Anti-melanogenic activity was confirmed via the intracellular melanin content, extracellular melanin content, a tyrosinase inhibitory assay, a Western blot assay, real-time PCR, and a cAMP assay using the ethyl acetate fraction. In addition, compounds included in the ethyl acetate fraction (EF) were confirmed through LC-MS/MS. In addition, the total polyphenol content, total flavonoid content, and total antioxidant activity of the *C. ovata* water extract and its fractions were measured to determine whether antioxidant activity affected the anti-melanogenic effect.

In this study, we investigated whether *C. ovata* and EF are able to inhibit melanogenesis and uncovered the molecular mechanisms underlying this effect.

## 2. Results

### 2.1. Yields and Antioxidant Activity of C. ovata Water Extract (COE) and Its Fractions

**Figure 1:**
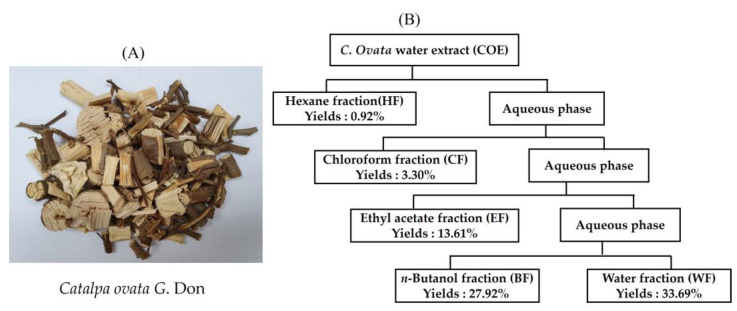
*Catalpa ovata G.* Don (**A**) and the scheme of the fractionation of the *C. ovata* water extract (COE) using organic solvent partitioning (**B**).

The extraction yield of the *C. ovata* water extracts (COEs) was 6.02%, and those of their fractions—HF, CF, EF, BF, and WF—were 0.92%, 3.30%, 13.61%, 27.92%, and 33.69%, respectively (Figure 1). The order of the total extraction yields of the COE fractions was as follows: HF < CF < EF < BF < WF.

Table 1 shows the total polyphenol and total flavonoid content in the COE and its fractions. Total phenolic (91.56 ± 2.81 mg GAE/g extract) and flavonoid (59.87 ± 0.52 mg CE/g extract) contents were present in significant amounts in the COE. Significant differences in the polyphenol and flavonoid contents were observed in the five fractions. In particular, EF contained higher polyphenol (169.54 ± 3.32 mg GAE/g extract) and flavonoid (134.12 ± 0.52 mg CE/g extract) contents than those of the COE and other fractions, respectively. However, the polyphenol (HF; 22.92 ± 0.29 mg GAE/g extract, WF; 23.06 ± 0.23 mg GAE/g extract) and flavonoid (HF; 51.09 ± 0.02 mg CE/g extract, WF; 1.69 ± 0.05 mg CE/g extract) contents of HF and WF were found to be lower than those of the COE.

The total antioxidant activity of the COE and its fractions was measured via the FRAP and ABTS assays (Table 1). Our comparative results for the COE and its fractions indicated that their total antioxidant capacities increased in the following order: WF < HF < COE < BF < CF < EF. The EF exhibited the most powerful antioxidant capacity among the COE and its fractions, and it was approximately two times greater than that of the COE.

### 2.2. Effect of C. ovata Extract (COE) and Its Fractions on Cell Viability, Melanogenesis, and Tyrosinase Activity

**Figure 2:**
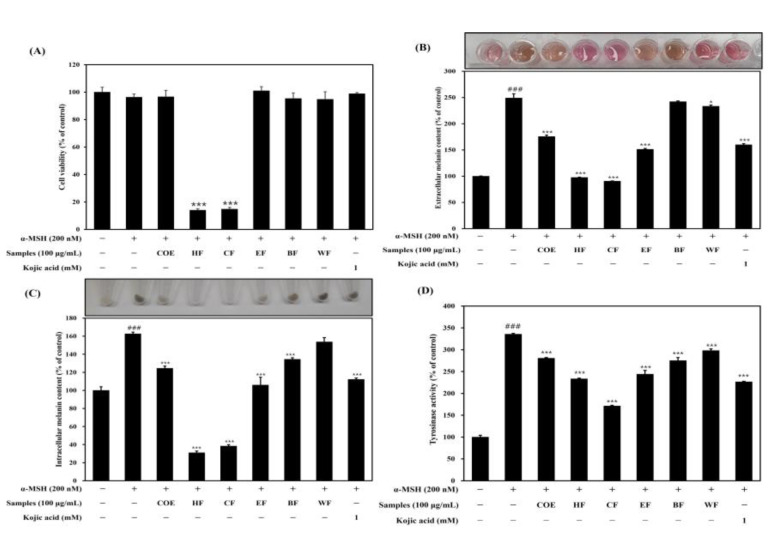
Effect of the *C. ovata* extract and its fractions on (**A**) cell viability, (**B**) extracellular melanin content, (**C**) intracellular melanin content, and (**D**) tyrosinase activity in α-MSH-stimulated B16F1 cells. Cells were treated with the *C. ovata* extract (100 μg/mL) and its fractions and kojic acid (1 mM, as positive control) with or without α-MSH (200 nM) for 72 h. COE, water extract; HF, hexane fraction; CF, chloroform fraction; EF, ethyl acetate fraction; BF, *n*-butanol fraction; WF, water fraction. The results were expressed as the mean ± SD from the three independent experiments. ### *p* < 0.001 compared with the control group; * *p* < 0.05 and *** *p* < 0.001 compared with the α-MSH-stimulated group.

The effect of the COE and its fractions on B16F1 cell viability was determined using an MTT assay. B16F1 melanoma cells were incubated with the COE and its fractions (at 100 μg/mL) with α-MSH (200 nM) for 72 h, and cell viability was tested. As shown in Figure 2A, the cell viability in the treatments with COE and its fractions was over 95%, and there was no significant effect compared to the non-treated cells. However, the cell viability of the hexane and chloroform fractions was significantly reduced to under 20%. Next, we investigated the effect of the COE and its fractions on α-MSH-stimulated melanogenesis, the quantity of intracellular melanin, the quantity of extracellular melanin, and tyrosinase activity. As shown in Figure 2B,C, the extra-/intracellular melanin contents were decreased via the treatment with the COE and its fractions (extracellular melanin content: control, 100.00 ± 0.57%; α-MSH, 249.14 ± 7.91%; COE, 175.61 ± 2.93%; hexane fraction (HF), 97.52 ± 0.65%; chloroform fraction (CF), 90.66 ± 0.32%; ethyl acetate fraction (EF), 151.23 ± 1.74%; *n*-butanol fraction (BF), 242.09 ± 1.31%; water fraction (WF), 233.33 ± 2.37%; kojic acid, 160.00 ± 1.97%; intracellular melanin content: control, 100.00 ± 4.06%; α-MSH, 162.66 ± 1.88%; COE, 124.46 ± 2.47%; HF, 30.94 ± 1.86%; CF, 38.29 ± 1.61%; EF, 105.89 ± 8.62%; BF, 134.33 ± 1.81%; WF, 153.57 ± 1.78%; kojic acid, 112.18 ± 1.46%). It can, thus, be confirmed that the COE and all of its fractions had inhibitory effects on melanin synthesis. The extracellular/intracellular melanin content was very low in the treatments with HF and CF. However, it seems that the melanin content was low due to the cytotoxicity of HF and CF. We found that EF significantly reduced the extra-/intracellular melanin content without cell toxicity. Our results indicated that EF suppressed the intracellular melanin content in a similar manner to that of the positive control (kojic acid, 1 mM).

We evaluated the tyrosinase inhibition effects of the COE and its fractions, and our results showed that α-MSH (335.52 ± 3.44%) increased the tyrosinase activity (Figure 2D). The treatments with the COE and its fractions decreased the tyrosinase activity (COE, 280.52 ± 3.28%; HF, 233.15 ± 41.40%; CF, 171.05 ± 9.51%; EF, 243.94 ± 8.24%, BF, 275.00 ± 4.62%; WF, 297.89 ± 7.94%) and kojic acid (226.31 ± 3.97%). The results showed that HF, CF, and EF displayed strong tyrosinase activity. Based on the cell viability results, HF and CF were excluded from the subsequent experiments. Out of all of the fractions, we selected EF for further analyses. 

### 2.3. Effect of EF on Cell Viability, Melanogenesis, and Tyrosinase Activity

**Figure 3:**
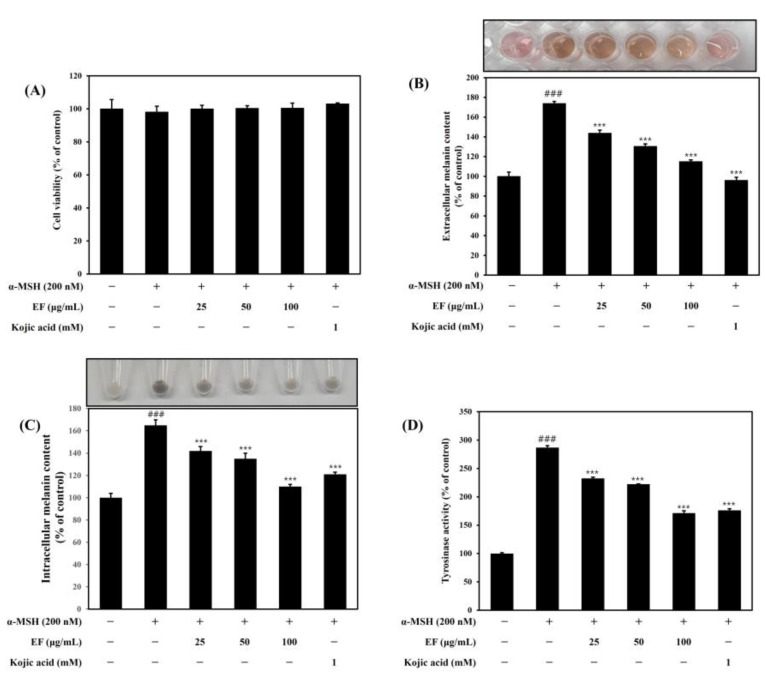
Effect of the ethyl acetate fraction (EF) of *C. ovata* extract on (**A**) cell viability, (**B**) extracellular melanin content, (**C**) intracellular melanin content, and (**D**) tyrosinase activity in stimulated B16F1 cells. Cells were treated with various concentrations of EF and kojic acid (1 mM) with or without α-MSH (200 nM) for 72 h. Kojic acid was used as a positive control. The results were expressed as the mean ± SD from the three independent experiments. ^###^ *p* < 0.001 compared with the control group; *** *p* < 0.001 compared with the α-MSH-stimulated group.

The melanin content and tyrosine activity of B16F1 cells after treatment with EF at various concentrations are shown in Figure 3. The results indicate that the extracellular content and intracellular melanin content in α-MSH-treated cells increased about 170% and 165% over those of the control cells. The positive control (kojic acid at 1 mM) and the sample had significantly reduced extracellular and intracellular melanin contents, respectively. EF inhibited the production of both extracellular and intracellular melanin contents in a-MSH-induced B16F1 cells in a dose-dependent manner. However, the intracellular melanin contents were 142.11 ± 4.10%, 135.20 ± 5.22%, and 110.32 ± 2.55% for the cells treated with the extract at 25, 50, and 100 µg/mL, respectively. Similarly to the melanin content analysis, a 280% increase in cellular tyrosinase activity was observed in the α-MSH-treated cells compared to the control cells. As shown in Figure 3D, EF inhibited cellular tyrosinase in a dose-dependent manner. Also, EF (at 100 μg/mL) similarly reduced cellular tyrosinase activity compared to that in the cells treated with kojic acid.

### 2.4. EF Inhibits α-MSH-Induced Melanogenesis through the Modulation of Melanogenesis-Related Protein Expression

**Figure 4:**
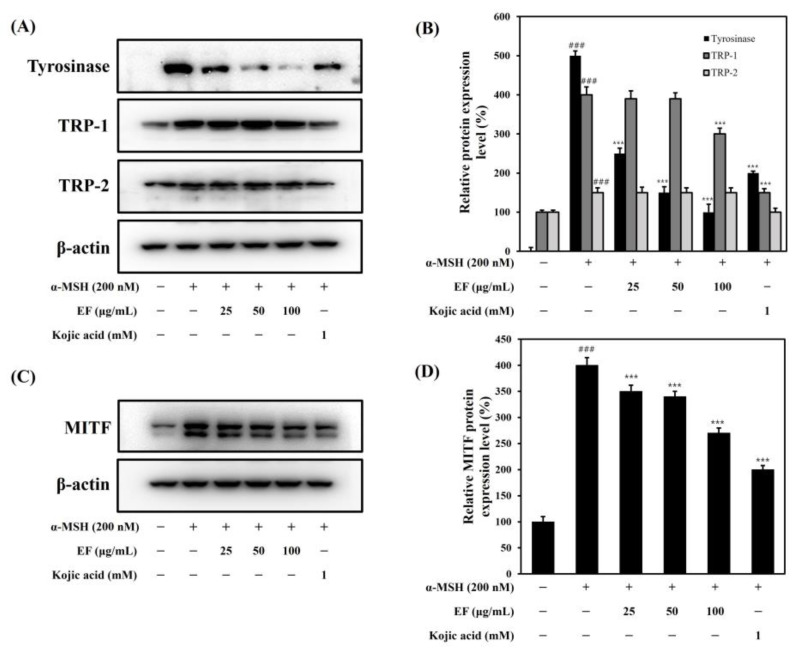
Inhibitory effect of EF on the expression of melanogenesis-related proteins in B16F1 cells. Cells were treated with various concentrations of EF, kojic acid, and α-MSH (200 nM) for 72 h (**A**,**B**) or 4 h (**C**,**D**). The results are expressed as the mean ± SD from three independent experiments. ^###^ *p* < 0.001 compared with the control group; *** *p* < 0.001 compared with the α-MSH-stimulated group.

To understand the effect of EF on the molecular mechanisms underlying α-MSH-induced melanogenesis, we examined the expression of several proteins related to melanogenesis using Western blot assays. The results showed that α-MSH increased the expression of MITF, tyrosinase, and TRP-1 and TRP-2 proteins (Figure 4). EF reduced the expression of tyrosinase in a dose-dependent manner (Figure 4). However, EF suppressed TRP-1 at 100 μg/mL only and did not affect the expression of TRP-2.

**Figure 5:**
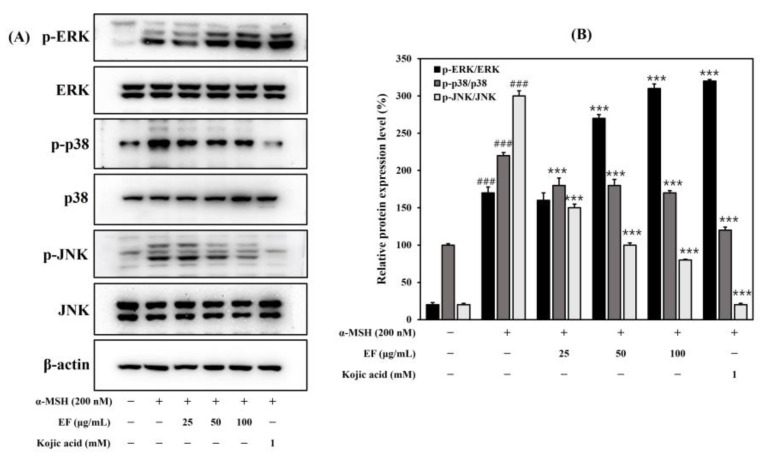
Inhibitory effect of EF on the expression of MAPKs (**A**,**B**) in B16F1 cells. Cells were treated with various concentrations of EF, kojic acid, and α-MSH (200 nM) for 30 min. The results are expressed as the mean ± SD from three independent experiments. ^###^ *p* < 0.001 compared with the control group; *** *p* < 0.001 compared with the α-MSH-stimulated group.

In the EF-treated group, the MITF expression decreased in a dose-dependent manner. Thus, we expected that the anti-melanogenic effect of EF was contributed by the downregulation of the melanogenic transcription factor MITF. The MAPK family consists of p38 MAPK, p42/44 MAPK, and c-Jun N-terminal kinase (JNK). We tested whether EF could downregulate melanin synthesis through MAPK (p38, ERK, and JNK) signaling pathways. Our results showed that the expression of phosphorylated p38 and JNK was increased after the α-MSH treatment, and the expression of the phosphorylation of p38 and JNK was inhibited in a dose-dependent manner via the EF treatment (Figure 5). However, the phosphorylation of ERK was increased in a dose-dependent manner with the EF treatment. The phosphorylation of ERK is known to induce a decrease in MITF, leading to ubiquitin-dependent proteasomal degradation [24]. Therefore, these results indicated that EF was involved in a reduction in melanogenesis through the regulation of the MAPK pathway. Taken together, these results suggest that EF suppresses MITF expression and melanin synthesis through the upregulation of ERK phosphorylation.

### 2.5. EF Inhibits α-MSH-Induced Melanogenesis through the Modulation of the Melanogenesis-Related mRNA Gene

**Figure 6:**
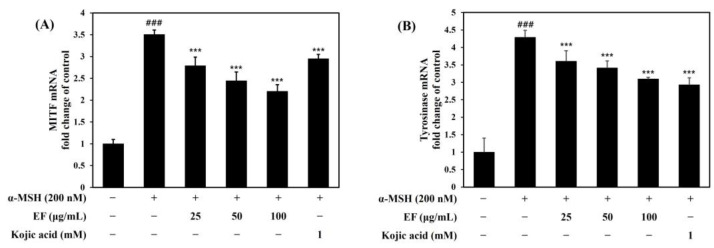
Inhibitory effect of EF on the expression of MITF mRNA (**A**) and tyrosinase mRNA (**B**) in B16F1 cells. Cells were treated with various concentrations of EF (0–100 μg/mL), kojic acid (1 mM), and α-MSH (200 nM) for 2 h (**A**) or 24 h (**B**). qRT-PCR was conducted to evaluate the MITF and tyrosinase mRNA levels. The results were normalized against the 36B4 expression. ^###^ *p* < 0.001 versus control; *** *p* < 0.001 versus α-MSH.

The *MITF* transcription factor has been recognized as an activator of tyrosinase mRNA expression [25,26], and the inhibition of *MITF* expression may decrease tyrosinase mRNA expression and reduce tyrosinase protein activity. As shown in Figure 6A,B, α-MSH increased the *MITF* mRNA and tyrosinase mRNA expression in B16F1 cells in a similar manner to that of the melanin content and tyrosinase activity. However, we observed that treatment with EF attenuated α-MSH-induced *MITF* mRNA and tyrosinase mRNA expression. These results indicate that EF has an anti-melanogenic effect on B16F1 mouse melanoma cells.

### 2.6. EF Reduces p-CREB and cAMP Concentrations

**Figure 7:**
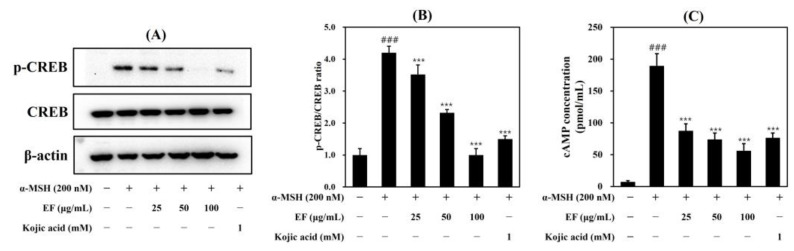
Effect of EF on p-CREB expression (**A**,**B**) and cAMP concentration (**C**) in stimulated B16F1 cells. Cells were treated with various concentrations of EF and kojic acid (1 mM) for 24 h and then α-MSH (200 nM) for 20 min. The results were expressed as the mean ± SD from three independent experiments. ^###^ *p* < 0.001 compared with the control group; *** *p* < 0.001 compared with the α-MSH-stimulated group.

Previous studies have reported that several signaling pathways are involved in melanogenesis, including a cAMP-mediated signaling pathway and an MAPK signaling pathway [27,28]. Therefore, to determine the inhibitory mechanisms of the EF-induced anti-melanogenic effect, we assessed the phospho-CREB protein level using a Western blot assay and the cAMP concentration via a cAMP assay for the cAMP signaling pathway. As shown in Figure 7, EF reduced the phospho-CREB and cAMP levels in a dose-dependent manner. This supports our suggestion that EF inhibits melanogenesis by degrading tyrosinase and downregulating melanogenic genes through the inhibition of cAMP signaling.

### 2.7. Identification and Quantification of Six Compounds in EF Using High-Performance Liquid Chromatography (HPLC)

**Figure 8:**
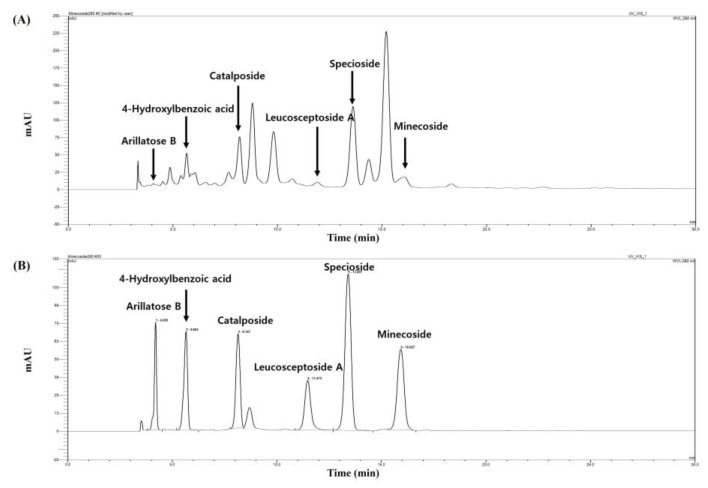
HPLC chromatogram of the EF (**A**) and standard mixture (**B**) at 280 nm. mAU: milli-absorbance. The mobile phase consisted of a mixture of solvents A (water containing 0.1% acetic acid) and B (acetonitrile), and a gradient elution (from 80:20 to 70:30, *v*/*v*) was employed for 30 min at a flow rate of 1.0 mL/min.

Among the several compounds predicted for EF using LC-MS/MS, six major compounds were confirmed using HPLC analysis (Figure 8A). The six major compounds were arillatose B, 4-hydroxybenzoic acid, catalposide, leucosceptoside A, specioside, and minecoside, and the peak retention times were found to be 4.20 min, 5.65 min, 8.14 min, 11.47 min, 13.40 min, and 15.92 min, respectively (Figure 8B). The contents of arillatose B, 4-hydroxylbenzoic acid, catalposide, leucosceptoside A, specioside, and minecoside in EF were 15.27 μg/mg, 19.51 μg/mg, 100.20 μg/mg, 72.32 μg/mg, 104.26 μg/mg, and 63.71 μg/mg, respectively. The EF showed high contents of catalposide, specioside, and minecoside.

## 3. Discussion

*C. ovata* is used as an anti-inflammatory drug in traditional Korean medicine. In previous studies, the antioxidative activity of *Catalpa* plant leaves and the antifungal activity of *C. ovata* stems have been reported [29,30]. A recent investigation of the bioactive components of *C. ovata* reported that the main compound, catalposide, had an anti-inflammatory effect on RAW264.7 macrophage cells and a protective effect against oxidative damage [31,32]. Additionally, several studies have also reported the cytoprotective effects of catalposide against oxidative damage in neuronal cells and the effects of catalpalactone on dopamine biosynthesis and _L_-DOPA-induced cytotoxicity [33,34]. It was reported that catapalactone, 9-hydroxy-ɑ-lapachone, and 4,9-dihydroxy-ɑ-lapachone exhibited an inhibitory effect on the production of nitric oxide [33]. Interestingly, it was reported that *p*-hydroxybenzoic acid from the branches of *Ficus erecta* var. sieboldii King had inhibitory activity against tyrosinase [35].

Although several studies on the efficacy of *C. ovata* have been performed, the molecular mechanism of its anti-melanogenic effect has not yet been elucidated.

In this study, the anti-melanogenic effects of *C. ovata* and EF were reported for the first time. We determined the influence of the COE and its fractions on the inhibitory effect on melanin synthesis in α-MSH-stimulated B16F1 cells. First, we tested the cytotoxicity of the COE and its fractions at a concentration of 100 μg/mL and found that there was no cytotoxicity, except in the hexane and chloroform fractions. However, Young et al. isolated lupeol, 2(4-hydroxyphenyl) ethyl triacotanoate, and a mixture of 9-hydroxy ɑ-lapachone, 9-mthoxy ɑ-lapachone, and ferulic acid from hexane and chloroform fractions of *C. ovata* [36]. The cytotoxicity of HF and CF is possibly related to their anticancer activity.

Our results showed that EF markedly inhibited α-MSH-induced melanin synthesis and tyrosinase activity in a dose-dependent manner. Next, we selected EF from the fractions and confirmed the molecular mechanisms of its anti-melanogenic effect.

Tyrosinase is known to be an essential rate-limiting enzyme for melanin synthesis [37]. Various melanogenesis-related proteins, such as tyrosinase, TRP-1, and TRP-2, are key mechanisms by which tyrosinase inhibitors regulate melanogenesis [38]. Therefore, it is well known that decreasing tyrosinase activity is associated with the inhibition of melanogenesis. Our results showed that *C. ovata* and EF decreased melanin content and tyrosinase activity.

Generally, α-MSH induces melanin synthesis through MITF expression, which is stimulated by tyrosinase. MITF is a transcription factor that binds to the promoters of tyrosinase—TRP-1 and TRP-2—and activates the transcription of genes required for melanin biosynthesis [39]. Therefore, it was important to downregulate the expression of MITF to confirm the inhibitory effect of EF on melanogenesis. Our results showed that EF significantly suppressed the mRNA and protein levels of MITF in α-MSH-stimulated B16F1 cells. Thus, these results indicated that the inhibitory effect of EF was accompanied by the corresponding downregulation of MITF and tyrosinase expression and, consequently, reduced melanogenic protein expression.

Several signaling pathways are involved in melanin synthesis, of which MAPK signaling is known to influence the melanin synthesis process. The regulation of MAPK families such as ERK, p38 MAPK, and JNK is involved in melanogenesis in melanocytes. The role of ERK in the MITF proteasome degradation pathway is that its active form phosphorylates MITF at serine-73 in post-translational processes and induces ubiquitination and proteasome degradation, ultimately reducing the synthesis of melanogenic proteins [40]. On the other hand, the phosphorylation of p38 and JNK can activate MITF, which may transcriptionally regulate the expression of tyrosinase, thus inducing melanin production [41]. Based on these studies, we observed that EF markedly decreased phosphorylated p38 and JNK and increased phosphorylated ERK. The results of this study indicated that α-MSH-induced inhibition of MITF and tyrosinase expression was mediated by the inhibition of phospho-p38, JNK, and the activation of ERK signaling pathways. So, we suggest that the regulation of MAPK signaling by EF is attributable to the result of the inhibition of melanin synthesis. In addition, we found that EF had an inhibitory effect on melanin content and tyrosinase activity by suppressing the cAMP signaling pathway in α-MSH-induced B16F1 cells.

In a previous report, Park et al. (2018) [42] isolated 10 single-compound ethyl acetate fractions of *C. ovata*, namely, 1-*O*-*p*-coumaroyl-β-d-glucopyranose, arillatose B, *p*-hydroxybenzoic acid, 6-*O*-(*E*)-feruloyl-α-glucopyranoside, catalposide, 6-*O*-*trans*-feruloyl-5,7-bisdeoxycynanchoside, dehydroacteoside, 6-*O*-*trans*-feruloyl-3β-hydroxy-7-deoxyrehamaglutin A, minecoside, and *p*-hydroxyphenyl ferulate.

In our study, the main compounds in EF were identified using LC-MS/MS. The data provided by the literature and available databases were cited and characterized. Nineteen compounds were detected, namely, 1-*O*-*p*-coumaroyl-β-d-glucopyranose, arillatose B, 6-O-(*E*)-feruloyl-α-glucopyranoside, 4-Methylumbelliferyl b-L-fucopyranoside, catalposide, verminoside, Verbascoside, 6-O-trans-feruloyl-5,7-bisdeocycynanchoside, dehydroacteoside, isoverbascoside, 6-O-trans-feruloyl-3β-hydroxy-7-deoxyrehamaglutin A, specioside, leucosceptoside A, minecoside, miconioside A, grandifloroside, martynoside, picroside II, and picroside III, and all known compounds are shown in Supplementary Figure S1. Although nineteen major compounds of EF were identified, it was not confirmed whether all of these compounds directly inhibited melanin. In a future study, we intend to prove whether any other ingredients in EF have direct whitening action. Among the nineteen compounds found in EF, six commercially available compounds were analyzed using HPLC, and their contents in EF were measured. In particular, the contents of three compounds (catalposide, specioside, and minecoside) were confirmed to be high. We identified six compounds from the ethyl acetate fraction of the *C. ovata* extract and examined their capabilities for the inhibition of melanin synthesis. So far, of the six examined compounds, we could not find any significant effects on melanin synthesis compared to EF. In this experiment, the main compound with a whitening effect was not identified. Also, the compounds with large peak areas at retention times of 8.58 min and 14.76 min could not be identified, but the main peaks and whitening efficacy will be confirmed through future experiments. 

Taking its results together, this study shows that EF has an excellent melanin inhibition effect, but this remains to be confirmed in normal human melanocytes and human skin.

## 4. Materials and Methods

### 4.1. Materials and Reagents

The alpha-melanocyte-stimulating hormone (α-MSH) and L-3,4-dihydroxyphenylalanine (L-DOPA) were purchased from Sigma-Aldrich (St. Louis, MO, USA). Antibodies for tyrosinase, TRP-1, and TRP-2 and secondary antibodies were obtained from Santa Cruz biotechnology (Santa Cruz, CA, USA), and antibodies for phospho-p38, phospho-JNK, phospho-ERK, MITF, and actin were obtained from Cell Signaling Technology (Beverly, MA, USA). In addition, *C. ovata* was obtained from Hanbang Herbal Drug Co. (Jecheon, Korea). All other reagents were of the highest commercially available grade.

### 4.2. Extract Preparation and Fractionation

Dried *C. ovata* (100 g) was extracted with distilled water (1.0 L) at 100 °C for 2 h. The water extract was filtered with Whatman No. 41 filter paper (Whatman International Limited, Kent, UK), and the filtered extract was concentrated until the extract was at 70% volume at 60 °C under −0.08 MPa using a rotary vacuum evaporator (N-1000, EYELA Co., Tokyo, Japan). The *C. ovata* water extracts were lyophilized using a vacuum freeze dryer (Il Shin Lab Co., Yangju, Korea). *C. ovata* was authenticated by Prof. K.H. Leem (Department of Herbology, Semyung University), and a voucher specimen (KUB19-20) was deposited in the Kokuk University Herbarium. The *C. ovata* water extract (COE, 5 g) was consecutively fractionated with *n*-hexane (HF, 500 mL × 3 times), chloroform (CF, 500 mL × 3 times), ethyl acetate (EF, 500 mL × 3 times), *n*-butanol (BF, 500 mL × 3 times), and distilled water (WF) (in that order) while using separatory funnels. Finally, the fractions were concentrated using a rotary vacuum evaporator and lyophilized using a vacuum freeze dryer.

### 4.3. Total Polyphenol Content and Total Flavonoid Content

The total polyphenol content in the samples was determined using a modified Folin–Ciocalteu method. Briefly, 0.2 mL of the sample was mixed with 0.1 mL of 1 N Folin–Ciocalteu reagent and incubated for 3 min. Sodium carbonate (20%, 0.3 mL) was added to the reaction mixture for 30 min, and color development was observed at 700 nm. The total polyphenol content was expressed as milligrams of gallic acid equivalents per gram of extract (mg GAE/g extract). The total flavonoid content in the samples was measured according to the following method. Samples (0.25 mL) were made up to 1.5 mL with DW. Sodium nitrite (5%, 75 μL) was added for 5 min, then mixed with AlCl_3_ (10%, 150 μL) and incubated for 6 min. NaOH (500 μL, 1 N) and DW (275 μL) were added sequentially. The resulting solution was shaken vigorously, and the absorbance was measured at 510 nm. The total flavonoid content in the samples was calculated from the calibration plot and expressed as milligrams of catechin equivalents per gram of extract (mg CE/g extract).

### 4.4. Total Antioxidant Activity Using the FRAP Assay and ABTS Radical Scavenging Activity

The ferric-reducing antioxidant power was measured according to a previously described method [43]. The FRAP reagent contained 10 mL of a 10 mM 2,4,6-tripyridyl-s-triazine (TPTZ) solution in 40 mM HCl, 10 mL of 10 mM FeCl3∙H2O, and 100 mL of 300 mM acetate buffer (pH 3.6). The FRAP reagent (1 mL) was mixed with 50 μL of sample or standard and incubated at 37 °C for 10 min. The absorbance was measured at 593 nm.

The FRAP values were calculated from the calibration plot and expressed as mmol FeSO_4_/g extract. The antioxidant capacity was measured using the ABTS decolorization assay. ABTS cation radicals were produced by reacting 10 mL of 7.4 mM ABTS solution with 10 mL of 2.6 mM potassium persulfate solution, which was then stored in the dark overnight at room temperature. This solution was diluted with water to an absorbance of 1.5 ± 0.020 at 734 nm before use. Samples (100 μL) were mixed with 1 mL of ABTS solution and allowed to stand in the dark for 10 min. TEAC values were expressed as mmol TE/g extract.

### 4.5. Cell Culture and Treatment

B16F1 mouse melanoma cells were obtained from the Korean Cell Line Bank (Seoul, Korea). Cells were cultured in Dulbecco’s modified Eagle’s medium (DMEM; Gibco Life Technologies, Carlsbad, CA, USA) supplemented with 10% fetal bovine serum and 1% penicillin/streptomycin in a 5% CO_2_ humidified incubator at 37 °C.

### 4.6. Cell Viability

The cell viability of the cultured cells was determined using an MTT assay. B16F1 cells (1 × 10^4^ cells) were cultured in 24-well plates and incubated for 24 h. Samples and α-MSH-treated cells were incubated for 72 h at 37 °C in an atmosphere containing 5% MTT solution and were added to each well and incubated for 2 h. Dimethyl sulfoxide (DMSO) was added to dissolve the formazan crystals. Absorbance was measured at 540 nm by using a microplate reader (Thermo Electron Corp, Waltham, MA. USA). The optical density of the formazan formed in the control cells was taken as 100% viability.

Cell viability (%) = treated (O.D.)/control (O.D.) × 100

### 4.7. Measurement of Cellular Melanin Content

The melanin content was measured using the method described in [44] with slightly modifications. B16F1 cells were treated with samples and α-MSH for 72 h. Extracellular melanin content was measured with the supernatant. The intracellular melanin content was measured after washing with cold PBS and dissolving in 1 N NaOH with 10% DMSO at 80 °C for 20 min. The melanin content was determined by measuring the absorbance at 475 nm using a microplate reader. The melanin content was expressed in comparison to a control of 100%.

### 4.8. Measurement of the Cellular Tyrosinase Activity 

The cellular tyrosinase activity was monitored as described previously with slight modifications [45]. Briefly, B16F1 cells were treated with EF for 72 h, and the tyrosinase activity was estimated by measuring the rate of the formation of dopachrome from L-DOPA as a substrate for tyrosinase. The cells were lysed via incubation at 37 °C for 15 min in lysis buffer and centrifuged at 13,000 rpm for 3 min to obtain the supernatant. The reaction mixture contained 0.1 M sodium phosphate (pH 7.4), L-DOPA (2 mg/mL), and a supernatant. After incubation at 37 °C for 1 h, the dopachrome was monitored by measuring the absorbance at 405 nm using microplate reader. The value of each measurement is expressed as a percentage change in the control.

### 4.9. Western Blot Analysis

B16F1 cells were treated with EF, and the cells were cultured and harvested using lysis buffer. Protein concentrations were then determined using the Bradford assay (Bio-Rad, Hercules, CA, USA), after which proteins in 15 μg of the sample were separated by 10–12% sodium dodecyl sulfate–polyacrylamide gel electrophoresis (SDS-PAGE). The proteins were then transferred to a PVDF membrane (GE Healthcare, Buckinghamshire, UK). The membrane was blocked with 5% skim milk and then incubated overnight with primary antibodies. All bands were visualized via horseradish-peroxidase-conjugated secondary antibodies using an enhanced HRP substrate (Milipore, Billerica, MA, USA). The immune blots were visualized using a Davinch-Chemi™ imaging system (Young Wha Scientific Co. Ltd., Seoul, Korea), and the densitometry data were normalized to β-actin.

### 4.10. Quantitative Real-Time Polymerase Chain Reaction (qRT-PCR)

The cells were treated with EF (0–100 μg/mL), kojic acid (1 mM), and α-MSH (200 nM) for the indicated time periods (MITF for 2 h, tyrosinase for 24 h). Total RNA was prepared from B16F1 cells treated with EF using TRIZOL reagent^TM^ (Invitrogen, Carlsbad, CA, USA) according to the manufacturer’s protocols. cDNA was synthesized using cDNA reverse transcription kits (Applied Biosystems, Forster City, CA, USA) in a 20 μL total reaction volume. Reverse transcription was performed by incubating the mixture at 25 °C for 10 min and 37 °C for 120 min, and the reaction was terminated at 85 °C for 5 min. Real-Time PCR Master Mix in a final volume of 20 μL was used. qRT-PCR was carried out using the LightCycler 96 (Roche Molecular Biochemicals, Mannheim, Germany). The PCR cycles consisted of initial denaturation at 95 °C for 30 s, followed by 40 cycles of 95 °C for 5 s, 60 °C for 10 s, and 72 °C for 15 s. The Δ cycle threshold method was used for the calculation of the relative differences in mRNA abundance, and the LightCycler Data were analyzed using the comparative ΔΔCt method and presented as fold changes in gene expression normalized to 36B. The primer sequences used are listed in Table 2.

### 4.11. cAMP Assay

The cAMP concentration was measured with cell lysate using the cAMP parameter kit (R&D system, MN, USA). B16F1 cells were cultured on a 60 mm dish (3 × 10^5^ cells/dish) and treated with EF for 24 h. After the treatment with α-MSH (200 nM), cells were lysed for 20 min, and the cAMP concentrations were measured according to the manufacturer’s protocols.

### 4.12. HPLC Analysis

We performed HPLC to determine and quantify a few compounds in EF. A Thermo Scientific (Ultimate 3000) HPLC system (Thermo Scientific, Germering, Germany) equipped with a UV detector and 20 µL injection loop was used. Chromatographic separation was carried out on Thermo Scientific synchronic C_18_ columns (250 × 4.6 mm i.d., 5 μm). The flow rate was 1.0 mL/min, and the wavelength for UV detection was set to 280 nm. Mobile phases were formed using 0.1% acetic acid in DW as eluent A and in acetonitrile (ACN) as eluent B. The initial elution solution was 20% B, followed by a linear gradient to 30% B from 0 to 30 min. The solvent was filtered through a 0.22 µm filter and degassed. The sample injection volume was 20 µL.

### 4.13. Statistical Analysis

Data were reported as the mean ± standard deviation for triplicate determinations. Analysis of variance (ANOVA) accompanied by Tukey’s tests (GraphPad Prism 5) was conducted to identify the significant differences between samples (*p* < 0.05).

## 5. Conclusions

In this study, we confirmed that the *C. ovata* water extract and its ethyl acetate fractions inhibited melanin synthesis. In particular, we discovered that EF had strong anti-melanogenic effects through the inhibition of tyrosinase and anti-melanogenic proteins, such as tyrosinase, MITF, MAPK, p-CREB, and the cAMP pathway. Also, our results suggested that the total phenolic and flavonoid contents of EF were higher than those of the other fractions, and this may have contributed to the improvement of the antioxidant-related anti-melanogenic effect. Taken together, these results indicate that *C. ovata* extract has a significant anti-melanogenic effect and can be used as a treatment for skin hyperpigmentation disease.

## Figures and Tables

**Table 1:** Total polyphenol content, total flavonoid content, and antioxidant activity of the *C. ovata* water extract and its fractions.

Samples	Total Polyphenol (mg GAE/g Extract)	Total Flavonoid (mg CE/g Extract)	FRAP Value (mmol FeSO_4_/g Extract)	TEAC Value (mmol TE/g Extract)
COE	91.56 ± 2.81	59.87 ± 0.52	1.15 ± 0.02	0.81 ± 0.01
HF	22.92 ± 0.29	51.09 ± 0.02	0.07 ± 0.01	0.05 ± 0.01
CF	146.81 ± 4.24	82.60 ± 0.52	1.95 ± 0.02	1.23 ± 0.01
EF	169.54 ± 3.32	134.12 ± 0.52	2.25 ± 0.05	1.32 ± 0.02
BF	137.95 ± 2.10	97.75 ± 1.05	1.68 ± 0.02	0.91 ± 0.02
WF	23.06 ± 0.23	1.69 ± 0.05	0.19 ± 0.01	0.18 ± 0.01

COE, *C. ovata* water extract; HF, hexane fraction; CF, chloroform fraction; EF, ethyl acetate fraction; BF, *n*-butanol fraction; WF, water fraction; GAE, gallic acid equivalent; CE, catechin equivalent; FRAP, ferric-reducing antioxidant power; TEAC, Trolox-equivalent antioxidant capacity.

**Table 2:** Primers used for qRT-PCR.

Gene	Sequence (5′ to 3′)
MITF-forward	ATGCTGGAAATGCTAGAATACAGT
MITF-reverse	ATCATCCATCTGCATGCAC
Tyrosinase-forward	CCTCCTGGCAGATCATTTGT
Tyrosinase-reverse	GGCAAATCCTTCCAGTGTGT
36B4-forward	TGGGCTCCAAGCAGATGC
36B4-reverse	GGCTTCGCTGGCTCCCAC

## Data Availability

The data used to support the findings of this study are included in the article.

## Supplementary Materials

The following supporting information can be downloaded at: https://www.mdpi.com/article/10.3390/ijms25010151/s1.
