# Supplementary material for: Ethyl Acetate Fraction from a Catalpa ovata G. Don Extract Inhibits ɑ-MSH-Induced Melanogenesis through the cAMP/CREB Pathway
Source: Int J Mol Sci. 2023 Dec 21;25(1):151. doi: 10.3390/ijms25010151 (PMC10778878; doi:10.3390/ijms25010151)
Supplement: Supplementary file 1 [file ijms-25-00151-s001.zip › ijms-2733005-supplementary.pdf]

(A)

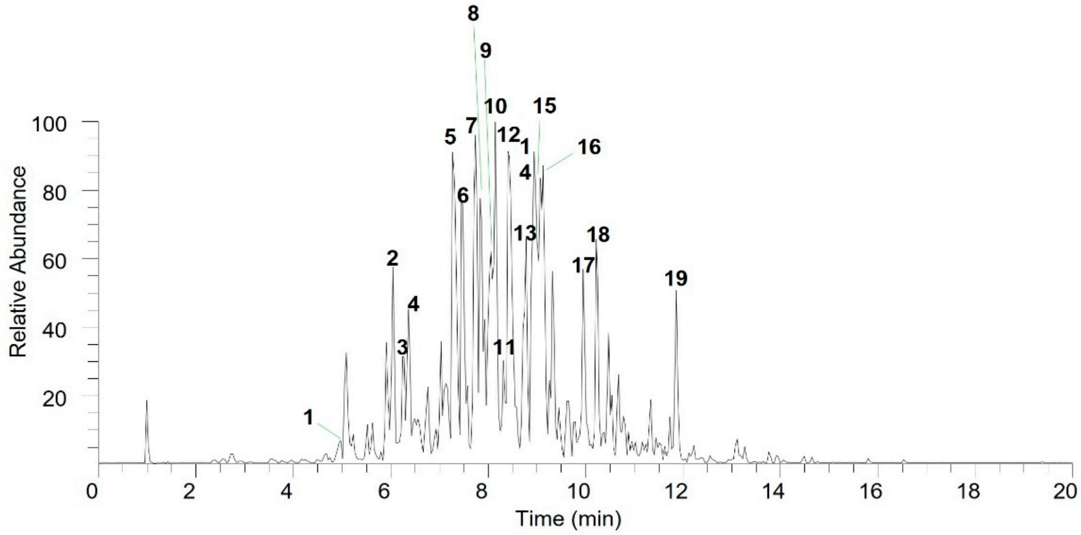

(B)

NO	RT(min)	m/z([M-H]-)	Formula([M-H]-)	Δ ppm	Compound
1	4.98	325.0931	$C_{15}H_{17}O_8$	3.987	1-O-p-coumaroyl-β-D-glucopyranose
2	6.04	517.1559	$C_{22}H_{29}O_{14}$	1.408	Arillatose B
3	6.24	355.1032	$C_{16}H_{19}O_9$	2.398	6-O-feruloyl-α-glucopyranoside
4	6.37	321.0981	$C_{16}H_{17}O_7$	3.833	4-Methylumbelliferyl b-L-fucopyranoside
5	7.26	481.1346	$C_{22}H_{25}O_{12}$	1.159	Catalposide
6	7.45	523.1451	$C_{24}H_{27}O_{13}$	0.885	Verminoside
7	7.74	623.1973	$C_{29}H_{35}O_{15}$	0.455	Verbascoside
8	7.87	539.1761	$C_{25}H_{31}O_{13}$	0.079	6-O-trans-feruloyl-5,7-Bisdeocycynanchoside
9	8.06	621.1821	$C_{29}H_{33}O_{15}$	1.084	Dehydroacteoside
10	8.15	623.1968	$C_{29}H_{35}O_{15}$	-0.332	Isoverbascoside
11	8.31	377.1241	$C_{19}H_{21}O_8$	2.694	6-O-trans-feruloyl-3β-hydroxy-7-Deoxyrehamag lutin A
12	8.41	507.1501	$C_{24}H_{27}O_{12}$	0.764	Specioside
13	8.72	637.2130	$C_{30}H_{37}O_{15}$	0.492	Leucosceptoside A
14	8.94	537.1608	$C_{25}H_{29}O_{13}$	1.048	Minecoside
15	9.07	607.2023	$C_{29}H_{35}O_{14}$	0.326	Miconioside A
16	9.13	537.1606	$C_{25}H_{29}O_{13}$	0.582	Grandifloroside
17	9.95	651.2281	$C_{31}H_{39}O_{15}$	-0.394	Martynoside
18	10.22	511.1450	$C_{23}H_{27}O_{13}$	0.671	Picroside II
19	11.86	537.1611	$C_{25}H_{29}O_{13}$	1.494	Picroside III

Supplementary Figure S1. LC-MS/MS analysis. (A) A representative LC-MS/MS chromatogram of EF. (B) Identification of the main components of EF.

LC-MS/MS conditions

LC/MS analyses were carried out using an LTQ Orbitrap XL (Thermo Fisher Scientific, Waltham, MA) coupled to an Accelar ultra-high-pressure liquid chromatography system (Thermo Fisher Scientific). The chromatographic separation of metabolites was achieved using a ACQUITY UPLC® BEH C18 column (2.1 × 150 mm, 1.7 μm) that was operated at 40 °C with mobile phases A (water with 0.1% formic acid) and B (acetonitrile with 0.1% formic acid). The solvent gradient conditions were as follows: 5% B at 0–1 min, 5–70% B at 1–20 min, 70–100% B at 20–24 min, and 100% B at 24–27 min. Each compound was detected with a photodiode array at 200–500 nm. The flow rate was 400 uL. For recycling, the initial gradient composition was restored and allowed to equilibrate for 3 min. The LC-MS system contained a heated electrospray ionization probe (HESI-II) as the ionization source. HESI was operated at 300 °C with a spray voltage of 5.0 kV. The nebulizer sheath and auxiliary gas flow rates were set to 50 and 5 arb, respectively. MS analysis was performed with polarity switching, and the following parameters were used for the MS/MS scan: m/z range of 100–1000; collision-induced dissociation energy of 45%;

data-dependent scanning mode. The Orbtrap analyzer was used for high-resolution mass-spectrum data acquisition with a mass-resolving power of 30,000 FWHM at m/z 400. The data-dependent tandem mass spectrometry (MS/MS) experiments were controlled using the menu-driven software provided with the Xcalibur system. All experiments were performed under automatic gain control conditions.
